# Plasmid-mediated antimicrobial resistance across One Health sectors: transmission dynamics and surveillance needs

**DOI:** 10.1128/msphere.00192-26

**Published:** 2026-07-17

**Authors:** Nuha Fairusya, Rongxuan Wang, Ryo Honda

**Affiliations:** 1 Graduate School of Natural Science and Technology, Kanazawa University 12858, Kanazawa, Japan; 2 Research Centre for Veterinary Science, National Research and Innovation Agency of Indonesia, Jakarta, Indonesia; 3 Faculty of Geosciences and Civil Engineering, Kanazawa University 12858, Kanazawa, Japan; 4 Center for Infectious Disease Education and Research (CiDER), Osaka University 13013, Suita, Japan; Shenzhen Institute of Synthetic Biology, Shenzhen, China

**Keywords:** antimicrobial resistance, environmental microbiology, plasmids, One Health

## Abstract

Antimicrobial resistance (AMR) is increasingly recognized as a One Health challenge driven by the continuous exchange of resistant bacteria and resistance determinants across human, animal, and environmental sectors. While genomic surveillance has substantially improved detection of antimicrobial resistance genes (ARGs), most monitoring frameworks remain gene- or isolate-centric, limiting insight into the mechanisms that govern resistance transmission and persistence. Recent evidence indicates that plasmids, self-replicating mobile genetic elements (MGEs) capable of horizontal transfer across bacterial species, play an important role in disseminating clinically relevant resistance determinants across sectors. In this mini-review, we synthesize genomic and ecological evidence demonstrating that a limited number of plasmid incompatibility (Inc) groups recur across human, animal, and environmental reservoirs, often independent of bacterial host lineages. We highlight how plasmid transmission dynamics are shaped by host-independent mobility, ecological generalism, co-selection with accessory traits, and persistence in engineered and natural environments. We further examine why current AMR surveillance approaches, including ARG-centric metagenomics and isolate-based monitoring, systematically overlook these plasmid-mediated processes. Furthermore, we propose that plasmid-resolved analysis represents a critical and currently underutilized complementary layer for One Health AMR surveillance. Integrating plasmid classification and genomic reconstruction into wastewater-based epidemiology and cross-sector monitoring frameworks can improve attribution of transmission pathways, enhance early detection of high-risk resistance, and provide a mechanistic foundation for risk-informed intervention strategies.

## INTRODUCTION

Antimicrobial resistance (AMR) is increasingly recognized as a One Health challenge driven by the continuous exchange of resistant bacteria and resistance determinants across human, animal, and environmental sectors. Previous AMR studies were more focused on antibiotic use in clinical and agricultural settings, but more recent studies have shown that the environment also plays an important role in AMR transmission dynamics. Global metagenomic surveys of urban sewage demonstrate that wastewater integrates resistance profiles shaped by human activity at regional and global scales, capturing clinically important antimicrobial resistance genes (ARGs) across diverse socio-economic contexts (1). Although municipal wastewater treatment reduces bacterial loads, treated effluents and episodic discharges, such as combined sewer overflows, continue to release large quantities of antimicrobial-resistant *E. coli* into receiving waters, positioning aquatic environments as major interfaces for AMR dissemination between sectors (2, 3).

Epidemiological evidence from One Health studies further underscores the permeability of boundaries between human, animal, and environment. Observational studies in low- and middle-income settings show that human colonization with extended-spectrum β-lactamase-producing *E. coli* is associated with animal contact and environmental exposures, while investigations of commercial swine and poultry systems report high burdens of antimicrobial-resistant *E. coli* across farms and abattoirs (4, 5). These findings establish cross-sector connectivity of AMR, but they provide limited insight into the genetic mechanisms that enable resistance determinants to move between hosts and environments. Current AMR surveillance frameworks remain focused on gene presence or abundance rather than the genetic vehicles responsible for dissemination (6). Culture- and PCR-based surveys of aquatic environments and metagenomic analyses of wastewater treatment systems consistently demonstrate widespread detection of clinically relevant resistance genes, including ESBLs, carbapenemases, and mobilized colistin resistance determinants (7, 8). However, global resistome analyses reveal that epidemiologically relevant, acquired ARGs exhibit strong geographic structuring, whereas latent ARGs are more globally dispersed and closely associated with bacterial community composition (9). Acquired ARGs are more frequently associated with mobile genetic elements (MGEs), and their uneven distribution reflects differences in gene mobilization and dissemination across ecological and anthropogenic contexts. This divergence indicates that AMR risk is not defined by the total resistome, but by the subset of ARGs that can be mobilized via MGEs.

Across wastewater treatment systems worldwide, ARG abundance correlates strongly with MGEs, with a substantial fraction of recovered genomes harboring putatively mobile resistance determinants (8). These observations highlight that identical ARGs may occur in fundamentally different genetic contexts with distinct mobility, host range, and epidemiological significance, rendering ARG abundance alone an incomplete proxy for transmission risk. While multiple MGEs contribute to AMR dissemination, plasmids are uniquely capable of autonomous replication and efficient horizontal transfer across bacterial species, enabling resistance traits to persist and circulate across ecological and sectoral boundaries. Increasing evidence demonstrates that the same plasmid lineages recur in human clinical isolates, livestock-associated bacteria, and environmental reservoirs, underscoring plasmid continuity within One Health networks (10, 11). Yet, plasmids remain largely invisible to routine surveillance, which continues to prioritize genes or indicator organisms rather than mobile genetic vehicles (12). This mini-review, therefore, argues that plasmid-resolved analysis represents a critical and currently underutilized complement to gene- and isolate-based AMR surveillance, providing insight into transmission dynamics that are otherwise obscured. Integrating plasmid-resolved analysis across human, animal, and environmental compartments is essential for understanding and predictive One Health surveillance.

## PLASMID GROUPS CIRCULATING ACROSS ONE HEALTH SECTORS

Plasmids are extrachromosomal DNA elements that play a crucial role in bacterial adaptation, gene transfer, and the dissemination of traits, such as AMR and virulence factors. Their classification is based on several genetic characteristics, environmental factors, evolutionary relationships, and functional properties. One of the primary methods used for classifying plasmid families is the PCR-based replicon typing (PBRT), which targets the replication regions of plasmids and determines their incompatibility (Inc) groups. Plasmids within the same Inc group cannot coexist in the same bacterial cell due to similar replication control mechanisms (13).

Early syntheses of plasmids harboring ARGs in *Enterobacteriaceae* established that AMR dissemination is structured by a limited number of recurrent plasmid families, with specific Inc groups repeatedly associated with characteristic resistance determinants and host ranges (14). These “epidemic plasmids” were recognized as independently evolving lineages whose distribution often transcends bacterial clonal backgrounds, providing a conceptual foundation for treating plasmids—not bacterial hosts alone—as meaningful units for understanding and monitoring AMR dissemination across sectors. Subsequent characterization of plasmids with ARGs in gram-negative bacteria revealed that successful plasmids frequently co-localize AMR, virulence, and metal tolerance genes and are stabilized by conserved replication, partitioning, and conjugation systems that promote persistence across diverse hosts (15). These features enable plasmids to function as semi-autonomous genetic entities whose evolutionary trajectories are shaped by both genetic architecture and ecological context, reinforcing their role in One Health AMR dissemination.

High-resolution plasmid epidemiology has since demonstrated that AMR spread is structured by non-random associations between ARGs and specific plasmid backbones. Detailed phylogenetic analyses of IncI1 plasmids show that a limited number of plasmid lineages carrying *bla*_CTX-M_ and *bla*_CMY_ genes have disseminated globally across humans, animals, and environmental reservoirs, frequently independent of bacterial host lineage (16). Similarly, large-scale analyses of complete plasmid sequences reveal recurrent global linkages between carbapenemase genes and particular Inc groups, such as IncL–*bla*_OXA-48_, IncX3/IncFII–*bla*_NDM_ variants, and IncFII/IncR–*bla*_KPC-2_, indicating that plasmid architecture constrains which resistance determinants persist and spread across sectors (17).

Recent environmental genomic studies further demonstrate that these epidemic plasmid families are not confined to clinical or agricultural settings (Table 1). Complete sequencing of extended-spectrum cephalosporin resistance plasmids from *E. coli* isolated in French rivers, for instance, identified a diverse but structured plasmidome dominated by IncI1, IncF, and IncN plasmids closely related to counterparts circulating in humans and food animals and detected across multiple *E. coli* phylogenetic backgrounds (18).

**TABLE 1:** Evidence for plasmid Inc groups associated with AMR across One Health sectors

Plasmid group	Bacterial host	ARGs	Source	Sector
IncA/C	*Enterobacter* spp. (19) *Escherichia coli* (20, 21) *Vibrio cholerae* (O1, O139) (22)	Aminoglycosides**:** *aadA3d*, *ant(3″)-IIa)*, *armA*, *rmtC* (20–22)	Clinical patient (19, 22) WWTP effluent (20)	Human (19, 21, 22) Environment (20, 22)
		Beta-lactams**:** *bla*_CTX-M-15_, *bla*_NDM-1_, *bla*_NDM-4_, *bla*_TEM-12_ (19–21)		
		Chloramphenicol**:** *floR* (22)		
		Macrolides**:** *mel*, *mph*(E), *mph*(K), *mphR*(A), *mph2*, *mrx*, *msr(E*) (20–22)		
		QACs**:** *qacE* (22)		
		Rifamycin**:** *Arr-2* (22)		
		Streptomycin***:*** *strA*, *strB* (22)		
		Sulfonamides**:** *sul1*, *sul2* (22)		
		Tetracycline**:** *tet*(A), *tetR*(A) (22)		
		Trimethoprim**:** *dfrA14* (22)		
IncF	*Citrobacter* spp. (19) *Enterobacter* spp. (19) *Escherichia coli* (18, 20, 21, 23–25) (ST131, ST205, ST405, ST1193, ST4204) (26) *Klebsiella oxytoca* (19) *Klebsiella pneumoniae* (23, 27)	Aminoglycosides**:** *aac (3)-IIe*, *aac (3)-IId*, *aac(6′)-Ib-cr5*, *aadA1*, *aadA2*, *aadA5*, *ant(3″)-IIa*, *aph(3′)-Ia*, *aph(3″)-Ib*, *aph(6)−1d* (20, 21, 24–28)	Pus, blood, urine from clinical patient (19, 26) Birds (21), swine (25) Animal abattoirs (23), river (18), seawater and sand (27), WWTP effluent (20), WWTP influent (28)	Human (19, 21, 26) Animal (21, 25) Environment (18, 20, 21, 23, 24, 27, 28)
		Beta-lactams**:** *bla*_CTX-M-1_, *bla*_CTX-M-3_, *bla*_CTX-M-14_, *bla*_CTX-M-15_, *bla*_CTX-M-27_, *bla*_CTX-M-32_, *bla*_CTX-M-55_, *bla*_CMY-2_, *bla*_DHA-1_, *bla*_HERA-3_, *bla*_NDM-5_, *bla*_NDM-7_, *bla*_OXA-1_, *bla*_SHV-12_, *bla*_SHV-134_, *bla*_TEM-1B_ (18–21, 23, 25–28)		
		Bleomycin**:** *bleO* (21)		
		Chloramphenicol**:** *c atA2*, *cmlA1*, *floR* (20, 21, 25–27)		
		Colistin**:** *mcr-9.1* (28)		
		Efflux pump**:** *sitABCD* (21)		
		Fosfomycin**:** *fosA3* (18)		
		Macrolides**:** *erm(B*), *mph*(A), *mph*(B) (26, 28)		
		QACs**:** *qacE*, *qacL* (25, 26)		
		Quinolones**:** *qnrB4*, *qnrS*, *qnrS2* (24–26)		
		Sulphonamides**:** *sul1*, *sul2*, *sul3* (20, 21, 24–27)		
		Tetracycline**:** *tet*(A), *tet*(B), *tet40* (20, 21, 24, 26, 28)		
		Trimethoprim**:** *dfrA12*, *dfrA14*, *dfrA17* (25, 26)		
IncHI1	*Citrobacter* spp. (19) *Klebsiella oxytoca* (19)	Beta-lactams**:** *bla*_CTX-M-1_, *bla*_CTX-M-3_ (19)	Clinical patient (19)	Human (19)
IncHI2	*Citrobacter* spp. (19) *Enterobacter* spp. (19) *Escherichia coli* (20, 21)	Aminoglycosides**:** *aac (3)-IIe*, *aac (3)-IV*, *ant(3″)-IIa*, *aph(3′)-Ia*, *aph(4)-Ia* (20, 21)	Clinical patient (19) Birds (21) WWTP effluent (20)	Human (19, 21) Animal (21) Environment (20)
		Beta-lactams**:** *bla*_CTX-M-9_, *bla*_CTX-M-15_, *bla*_CTX-M-55_, *bla*_CTX-M-65_, *bla*_NDM-1_, *bla*_NDM-5_, *bla*_TEM-32_, *bla*_TEM-122_ (19–21)		
		Chloramphenicol**:** *floR* (21)		
		Quinolones**:** *OqxA*, *OqxB* (21)		
		Sulfonamides**:** *sul1*, *sul2* (20, 21)		
		Trimethoprim**:** *dfrA1* (20)		
IncI complex	*Citrobacter* spp. (19) *Escherichia coli* (18, 20, 21)	Aminoglycosides**:** *aac (3)-VIa, ant(3″)-Ia*, *aadA5*, *aph(3′)-Ia* (18, 21)	Clinical patient (19) Birds (21) River (18) WWTP effluent (20)	Human (19) Animal (21) Environment (18, 20)
		Beta-lactams**:** *bla*_CTX-M-1_, *bla*_TEM-1_ (18–20)		
		QACs**:** *qacE* (21)		
		Sulfonamides**:** *sul1*, *sul2* (18, 21)		
		Tetracycline**:** *tet*(A) (18, 21)		
		Trimethoprim**:** *drfA17* (18)		
IncN	*Citrobacter* spp. (19) *Escherichia coli* (18, 20, 21, 29) *Klebsiella oxytoca* (19)	Aminoglycosides**:** *aac(6’)-Ib-cr, aadA16* (20, 29)	Clinical patient (19) Birds (21) River (18), WWTP biosolid (29), WWTP effluent (20)	Human (19) Animal (21) Environment (18, 20, 29)
		Beta-lactams**:** *bla*_CTX-M-1_, *bla*_CTX-M-3_, *bla*_CTX-M-15_, *bla*_OXA-1_, *bla*_TEM-1_ (18, 19, 29),		
		Quinolones**:** *qnrB17*, *qnrS1* (18, 20, 29)		
		Rifamycin**:** *Arr-3* (20)		
		Sulfonamides**:** *sul2* (29)		
		Tetracycline**:** *tet*(A) (20, 29)		
		Trimethoprim**:** *drfA14*, *drfA27*, *fosA3* (20, 29*)*		
IncP	*Escherichia coli* (29)	Aminoglycosides**:** *aadA6*, *aph(6)-Ib*, *aph(3″)-Id* (29)	WWTP biosolid (29)	Environment (29)
		Beta-lactams**:** *bla*_OXA-2_ (29)		
		Macrolides**:** *mph*(E), *msr*(E) (29)		
		QACs**:** *qacE* (29)		
		Sulfonamides**:** *sul1*, *sul2* (29)		
		Tetracycline**:** *tet*(A) (29)		
		Trimethoprim**:** *drfB1* (29)		
IncQ	*Enterobacter* spp. (19)	Beta-lactams**:** *bla*_CTX-M-15_ (19)	Clinical patient (19)	Human (19)
IncR	*Escherichia coli* (21) *Klebsiella pneumoniae* (27) *Salmonella enterica* ser. Wandsworth (30)	Aminoglycosides**:** *aac(6′)-Ib-cr*, *aph(3′)-IIa*, *aph(6)-Ic* (21, 30)	Fecal from clinical patient (30) River snail (30) Seawater and sand (27)	Human (30) Animal (21) Environment (27)
		Beta-lactams**:** *bla*_DHA-1_, *bla*_OXA-1_ (27, 30)		
		Chloramphenicol**:** *catB3* (30)		
		Macrolides**:** *mph*(A) (30)		
		Quinolones**:** *qnrB1* (27)		
		Rifamycin**:** *Arr-3* (30)		
		Sulfonamides**:** *sul1* (27, 30)		
IncU	*Aeromonas* spp. (31) *Citrobacter* spp. (19) *Escherichia coli* (29)	Aminoglycosides**:** *aac(6′)-Ib-cr5* (29)	Clinical patient (19) Hospital effluent-receiving water bodies (31), WWTP biosolid (29)	Human (19) Environment (29, 31)
		Beta-lactams**:** *bla*_CTX-M-3_, *bla*_KPC-2_, *bla*_OXA-1_, *bla*_OXA-473_, *bla*_OXA-513_ (19, 29, 31)		
		Chloramphenicol**:** *catB3* (29)		
		Efflux pump**:** *Mex* (31)		
		Macrolides**:** *mph*(A), *mrx*(A), *mph*(R) (29)		
		Quinolones**:** *qnrS2* (29)		
		Rifamycin**:** *Arr-3* (29)		
		Tetracycline**:** *tet*(A) (31)		
		Vancomycin**:** *van*(A) (31)		
IncX	*Escherichia coli* (21) *Citrobacter* spp. (19). *Klebsiella aerogenes* (19)	Aminoglycosides**:** *aph(3′)-Ia* (21)	Tissue, urine from clinical patient (19)	Human (19, 21)
		Beta-lactams**:** *bla*_CTX-15_, *bla*_NDM-1_, *bla*_NDM-5_, *bla*_SHV-12_ (19, 21)		
Col-type	*Citrobacter* spp. (19) *Enterobacter* spp. (19) *Klebsiella oxytoca* (19)	Beta-lactams**:** *bla*_CTX-M-9_, *bla*_CTX-M-14_, *bla*_CTX-M-15_, *bla*_CTX-M-27_, *bla*_CTX-M-55_, *bla*_CTX-M-65_ (19)	Clinical patient (19)	Human (19)

Among these studies, a limited number of plasmid Inc groups repeatedly emerged as major carriers of ARGs across the One Health sectors (Table 1). IncF plasmids were the most widely distributed and genetically diverse group detected in *E. coli*, *Klebsiella pneumoniae*, *Citrobacter* spp., and other *Enterobacterales* from clinical samples, livestock, birds, abattoirs, rivers, seawater, and wastewater systems. These plasmids carried a broad spectrum of AMR determinants, particularly ESBL, carbapenemases, aminoglycoside, quinolone, sulfonamide, tetracycline, and occasionally colistin resistance genes, highlighting their role as versatile, multi-replicon resistance scaffolds. IncA/C and IncHI2 plasmids also showed broad host ranges and multidrug resistance profiles, including carbapenemase and aminoglycoside resistance genes, and were recovered from clinical, animal, and wastewater-associated sources. IncI complex and IncN plasmids were frequently associated with ESBLs and quinolone resistance genes across rivers, birds, wastewater, and clinical isolates, suggesting their role in environmental persistence and dissemination. Smaller plasmid groups, such as IncP, IncQ, IncR, IncU, IncX, and Col-type plasmids, were detected less frequently but contributed to specific resistance contexts, including hospital effluent-associated carbapenem resistance (IncU), integron-linked resistance clusters (IncR), and clinical ESBL carriage (Col-type). Collectively, the reviewed studies demonstrate that while numerous plasmid Inc groups are present across One Health sectors, a core subset—particularly IncF, IncA/C, IncHI2, IncN, and IncI—dominates the epidemiology of plasmid-mediated AMR, with recurring detection in both clinical and environmental reservoirs. These studies indicate that a limited set of plasmid families characterized by distinct ARGs cargo, host ranges, and stabilization mechanisms recur across human, animal, and environmental compartments. This structured plasmidome provides a mechanistic basis for cross-sector AMR connectivity and motivates plasmid-centric frameworks for surveillance and risk prioritization.

## TRANSMISSION DYNAMICS OF PLASMID-MEDIATED AMR

Antimicrobial resistance dissemination is driven by a hierarchy of interacting MGEs, including insertion sequences, transposons, integrons, and plasmids, which together enable rapid recombination and horizontal gene transfer. Within this mobilome, plasmids are unique in their capacity for autonomous replication and efficient conjugative transfer, allowing them to function as self-transmissible platforms that integrate and disseminate diverse resistance modules across bacterial species and ecological compartments (32). Plasmid transmission dynamics are frequently decoupled from bacterial host population structure. A large multicountry genomic analysis of *E. coli* revealed that direct clonal transmission between humans, livestock, food, and environmental compartments was rare, whereas conserved IncI1 plasmids carrying extended-spectrum cephalosporin resistance were widely shared across sectors despite pronounced host phylogenetic divergence (33). Similarly, city-scale genomic analyses integrating human-associated wastewater, animal-associated waters, and environmental sites identified extensive cross-sector sharing of plasmids and ARGs, with many plasmids transferring independently of clonal strain spread and confirmed experimentally to be functionally transmissible across ecological boundaries (24).

Beyond transmission events, plasmid persistence is shaped by ecological interactions and selection pressures operating in both engineered and natural environments. Ecological network analyses of wastewater microbial communities demonstrate that plasmids carrying ARGs interact with significantly more bacterial hosts than non-AMR plasmids, acting as generalist connectors that increase network connectivity and resilience (34). Analyses of plasmids from wastewater treatment plant effluents reveal that plasmids commonly exist as co-resident communities, enabling plasmids to persist through co-selection with multidrug-resistant partners and stabilizing resistance determinants, even in the absence of strong antibiotic selection (20). A recent study that integrated plasmidomic, metagenomic, and whole-genome sequencing analyses of wastewater systems further shows that plasmids carrying multiple ARGs confer resistance to diverse antibiotic classes, promoting plasmid persistence under selective pressure and increasing the likelihood of maintenance across fluctuating environmental conditions (35).

Plasmid-mediated persistence also enables cryptic dissemination of resistance under conditions of low bacterial abundance or limited clonal expansion. An investigation targeting low-abundance ESBL-producing *Enterobacterales* demonstrated that plasmid-borne resistance determinants can persist and re-emerge across human, animal, and environmental compartments despite minimal host population expansion, highlighting transmission dynamics that are likely to be overlooked by an isolate-centric surveillance approach (19). In parallel, large-scale genomic analyses have identified the transmissible locus of stress tolerance (tLST) as a plasmid-associated element frequently co-localized with resistance genes in high-risk ESKAPE-E lineages, conferring tolerance to disinfectants, heat, and metals and thereby promoting plasmid persistence and co-selection across different settings (36). The convergence of host-independent transmission, ecological generalism, and co-selection mechanisms underscores plasmid-mediated gene transfer as the dominant process underpinning AMR connectivity across One Health systems.

## NEEDS OF ONE HEALTH GENOMIC SURVEILLANCE FRAMEWORK FOR UNDERSTANDING PLASMID-MEDIATED AMR DYNAMICS

Rapid advances in genomic technologies have enabled AMR genomic research in the environment, highlighting the important role of mobile genetic elements (MGEs) in the dissemination of ARGs across One Health sectors. However, the underlying dynamics remain poorly understood. Large-scale wastewater metagenomic surveys show that ARG abundance and composition vary widely across systems and are frequently decoupled from their mobile genetic context, limiting the ability of ARG-centric metrics to infer transmission risk or persistence potential (37). Similarly, shotgun metagenomic studies integrating resistome and mobilome profiling demonstrate that ARG abundance and mobility potential are often uncoupled, and that even comprehensive MGE analyses rarely resolve plasmid lineages or their cross-sector dissemination (38). As a result, current metagenomic surveillance frameworks provide limited capacity to distinguish highly mobile, high-risk resistance from environmentally persistent but epidemiologically less relevant gene pools (39). Isolate-based surveillance introduces additional blind spots by conflating resistance detection with bacterial host identity. Comparative analyses of *Acinetobacter* isolates from patients, livestock waste, and environmental waters reveal that ARGs are differentially localized on plasmids versus chromosomes across One Health sectors, with animal-associated isolates showing a greater reliance on plasmid-borne resistance than clinical or environmental counterparts (40). These findings underscore that ARG presence alone obscures sector-specific transmission strategies and highlight the limitations of surveillance approaches that fail to resolve whether resistance is carried on mobile plasmids or chromosomally fixed within particular hosts.

One Health AMR monitoring initiatives increasingly emphasize harmonized surveillance across human, animal, and environmental matrices, frequently prioritizing *E. coli* and ESKAPE pathogens as practical indicator organisms (41). While such frameworks provide critical guidance, they largely leave plasmid lineages and mobility traits unresolved. A similar limitation characterizes wastewater-based epidemiology, which is widely recognized as a cornerstone of One Health AMR surveillance due to its ability to integrate population-level signals from multiple sectors (42). Together, these blind spots indicate that technical advances alone are insufficient to improve AMR surveillance without a conceptual shift in the unit of analysis. By focusing on genes or indicator organisms rather than plasmid lineages and their ecological behavior, current surveillance systems systematically underestimate the mobility, persistence, and cross-sector connectivity that define plasmid-mediated AMR dissemination.

Recent AMR risk frameworks that prioritize gene mobility, host association, and ecological context implicitly point toward plasmids as key determinants of transmission risk, yet underutilized plasmid-resolved analysis (10). As a result, surveillance efforts continue to focus on ARGs or indicator organisms, despite growing evidence that plasmid lineages can capture the mobility, persistence, and cross-sector connectivity that define epidemiologically relevant AMR dissemination. A complementary plasmid-centric surveillance framework would explicitly treat plasmid lineages as the units of analysis, integrating hybrid whole-genome sequencing, plasmid reconstruction, and comparative genomics across human, animal, and environmental compartments. Within such a framework, plasmids could be risk-ranked based on features directly relevant to transmission, including conjugative capacity, host range, ecological persistence, and co-selection with accessory traits, such as metal tolerance or disinfectant resistance. Embedding plasmid-resolved data into wastewater-based epidemiology and One Health monitoring networks would enable early detection of high-risk plasmid lineages, improve attribution of cross-sector transmission pathways, and provide a mechanistic foundation for targeted intervention strategies.

**Fig 1:**
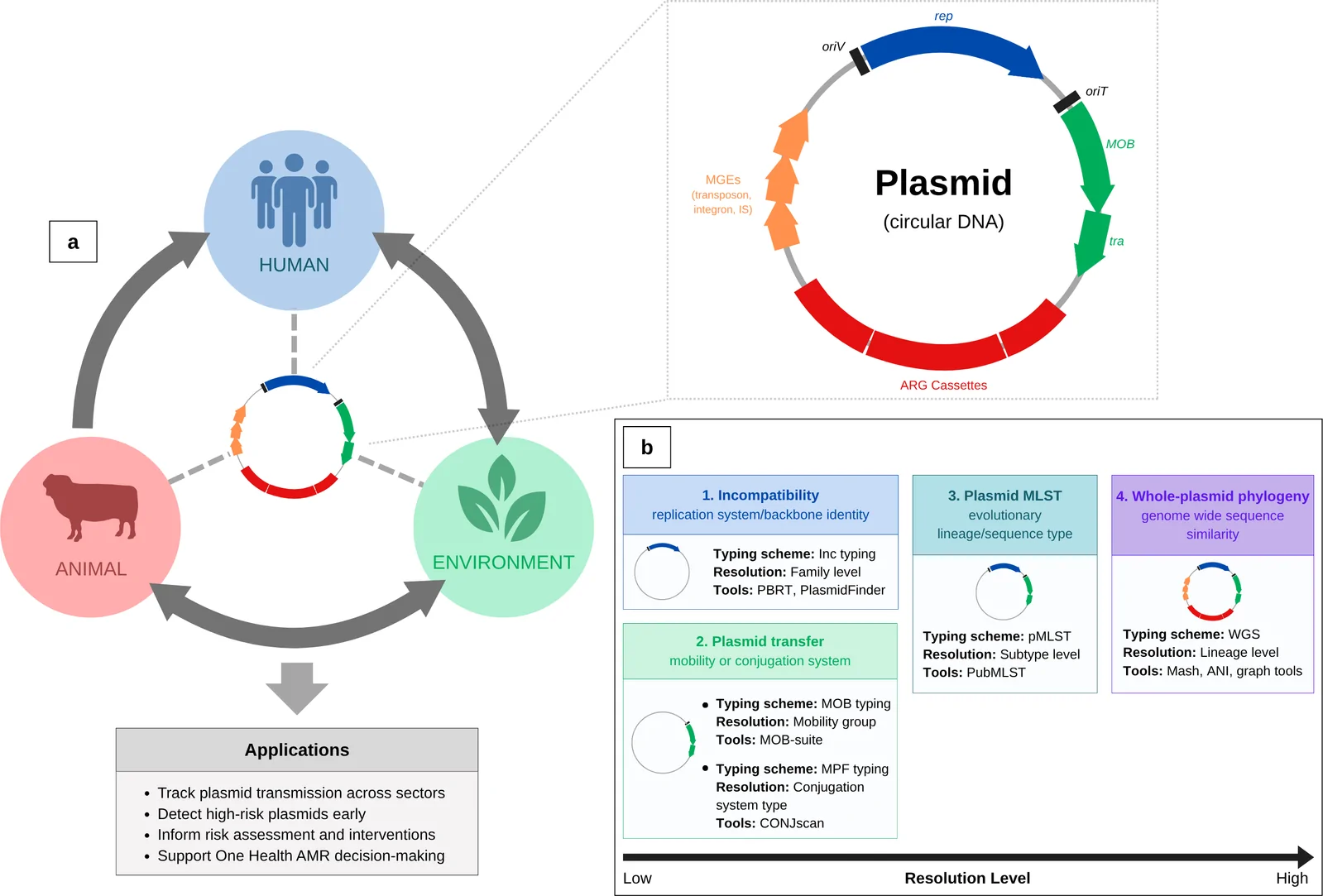
Current typical plasmid typing. (**a**) Integrating plasmid typing schemes using the One Health approach to understand plasmid-mediated AMR. (**b**) Plasmid typing scheme, resolution, and tools typically used. From left to right, the resolution level of the plasmid typing schemes is increased. (Note: pMLST scheme classifies plasmids based on the sequence variation of conserved backbone genes, which can include replication genes, partitioning genes, conjugation genes, and maintenance genes.)

Plasmid typing schemes classify plasmids at different biological and epidemiological levels, and each provides complementary information relevant to AMR surveillance (Fig. 1). Incompatibility typing based on replication systems remains the most widely used framework in AMR studies because it enables rapid family-level classification and cross-study comparability (43, 44). It is particularly valuable in One Health contexts where recurrent backbones, such as IncF, IncA/C, IncHI2, and IncN, appear across human, animal, and environmental compartments (Table 1). However, Inc typing alone does not indicate transfer potential or evolutionary relatedness. Mobility-based schemes, such as MOB typing and mating pair formation (MPF) classification, address this limitation by identifying relaxase genes and conjugation machinery, thereby predicting whether plasmids are mobilizable or self-transmissible (45). Higher-resolution methods, such as plasmid MLST (pMLST) and replicon sequence typing (e.g., the FAB formula for IncF plasmids), allow lineage-level tracking within major Inc groups and are particularly useful for outbreak investigations and cross-sector comparisons. Finally, whole-plasmid phylogeny or genome-wide similarity clustering provides the highest resolution by assessing overall sequence relatedness, capturing mosaic structure and recombination events that cannot be inferred from single-gene typing approaches (46).

Importantly, plasmid lineage alone does not define epidemiological risk. While typing frameworks, such as Inc classification, mobility typing, and pMLST, provide essential descriptors of plasmid identity and evolutionary relationships, risk is determined by the interaction between resistance gene cargo, mobility potential, host range, and ecological persistence (10, 32). Plasmids belonging to the same Inc group may differ substantially in ARG content or transfer capability, while unrelated plasmid lineages may converge in risk profile through acquisition of similar ARG combinations (11, 14). Ecological studies demonstrate that plasmids vary in host connectivity and persistence potential, influenced by co-selection, accessory adaptive traits, and environmental selection pressures (34, 47). Consequently, plasmid typing should be interpreted as a foundational layer within a broader risk-based framework integrating mobilome context, resistance gene composition, and epidemiological distribution across sectors.

Integrating these plasmid typing schemes into One Health surveillance frameworks can substantially improve the detection and interpretation of cross-sector transmission pathways. Incompatibility typing enables harmonized reporting across clinical, veterinary, and environmental laboratories, facilitating identification of recurring plasmid backbones that act as epidemiological connectors. MOB and MPF typing add functional insight by distinguishing plasmids with high dissemination potential from those that are likely to persist locally. Sequence-based subtyping and whole-plasmid phylogenetics further enhance resolution, allowing differentiation between truly shared plasmid lineages and unrelated plasmids that merely belong to the same Inc group. By combining backbone classification, mobility prediction, and evolutionary relatedness, plasmid typing can improve surveillance to understand how resistance circulates across humans, animals, and environmental reservoirs. This layered approach strengthens the One Health framework by identifying not only where ARGs occur, but which plasmid vehicles enable their persistence and spread across ecological boundaries.

## CONCLUSION

Growing genomic and ecological evidence demonstrates that plasmids play an important role in shaping the transmission, persistence, and cross-sector connectivity of AMR within One Health systems. Across diverse hosts and environments, a limited set of plasmid families repeatedly emerges as key vehicles of clinically relevant resistance, often decoupled from bacterial clonal expansion and sustained through ecological versatility and co-selection. These dynamics are poorly captured by surveillance approaches that focus exclusively on ARGs or indicator organisms. This minireview argues that plasmid-resolved analysis offers a complementary perspective for AMR surveillance by revealing transmission potential, host range, and persistence mechanisms that remain obscured. While technical and logistical challenges remain, recent advances in genomic sequencing and plasmid reconstruction increasingly enable integration of plasmid-level information into wastewater-based epidemiology and One Health monitoring networks. Incorporating plasmid classification and comparative genomics alongside established surveillance approaches can strengthen mechanistic understanding of AMR dissemination, improve risk prioritization, and support more informed intervention strategies across human, animal, and environmental domains.
